# Supplementary material for: Assessing Zymoseptoria tritici Candidate Effectors in the Non‐Host Nicotiana benthamiana Reveals Immune Recognition and Potential Host‐Independent Activities
Source: Mol Plant Pathol. 2026 Oct 30;27(11):e70344. doi: 10.1111/mpp.70344 (PMC13720751; doi:10.1111/mpp.70344)
Supplement: Supplementary file 1 [file MPP-27-e70344-s001.docx]

**Supplementary Figure 2.** Structural alignments of the Hce domain-containing effectors Mycgr3107904, Mycgr3111636 and Mycgr3104404.
